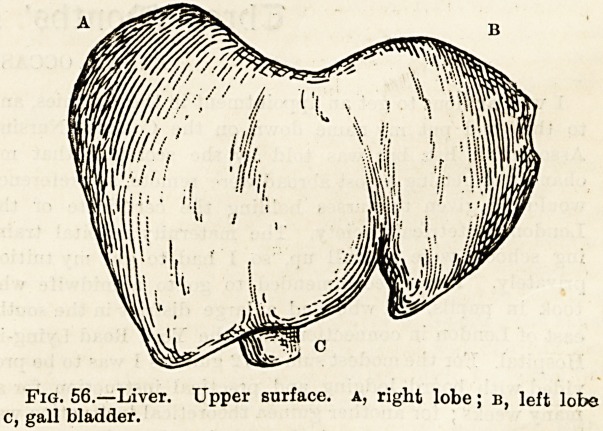# The Hospital. Nursing Section

**Published:** 1902-08-23

**Authors:** 


### The Hospital.

### Nursing Section.

Contributions for this Section of "THE HOSPITAL" should be addressed to the EDITOR, "THE HOSPITAL" NURSING SECTION, 28 & 29 Southampton Street, Strand, London, W.C.

No. 830.—VOL. XXXII. *SATURDAY, AUGUST* 23, 1902.

### Notes on News from the Nursing World.

### THE ROYAL RED CROSS.

THE King has been pleased to confer the decoration of the Royal Red Cross upon Miss H. Wedgwood, matron of the Royal Free Hospital, in recognition of her services in connection with the Army Nursing Reserve during the war in South Africa. The honour bestowed by his Majesty upon Miss Wedgwood will be greatly appreciated not only by the nurses of the school of which she is the esteemed head, but also by all who know how warmly she has at heart the interests of nursing generally, and how freely she has devoted time and trouble to the promotion of them.

### A NOTABLE MEMORIAL TABLET.

LAST week Lord Penrhyn unveiled, in St. David's Cathedral, a handsome brass tablet to the memory of the six members of the Welsh Hospital who died during the Boer War, two of whom were Miss Marion Lloyd, the matron, and Miss F. L. Sage, a nursing sister. Over the tablet was placed the hospital flag, which bears the Welsh dragon. The memorial was dedicated by the Bishop of St. David's, and Sir John Williams, chairman of the hospital committee, committed it to the charge of the Dean and Chapter. Lord Penrhyn, who delivered an address from the lectern, paid a warm tribute to the late Professor Hughes and the staff of the hospital, and said that while it was sad that those whose names they were commemorating had not lived to enjoy the triumph and welcome with which the return of the Hospital had been acclaimed, consolation might be derived from the thought that they had received their reward from the Giver of all good.

### NURSES FROM SOUTH AFRICA.

THE *Aurania*, which is due August 25th, has on board Sisters K. Webb, A. B. Noble, G. Deacon, L. E. Colston, M. E. Richardson, F. M. Learmouth, E. Johnson, V. P. Squire, E. McIntosh, A. A. Bonsfield, E. M. Renden, B. E. Caws, M. A. C. Millington, D. E. J. Wetton, E. Ellis, E. Prangley, A. E. Byrne, E. A. S. Webster, C. E. A. Thorpe, E. A. Chaffey, M. J. Mill, F. H. Barry, M. Ounsworth, E. M. Beetham, M. M. Tunley, G. K. Swanton, H. H. Mason, E. Kitching, P. M. Barsdorf, M. Talbot, and A. Austin. The *Braemar Castle*, due August 28th, has on board Sisters S. Clarke, A. McLeod, M. C. Reilly, A. M. Lashwood, H. Wolford Hansen, C. Cruickshank, and E. Smith.

### THE NIGHTINGALE FUND.

IN the report for the year 1901 just issued by the committee of the Nightingale Fund reference is made to the resignation of Miss Gordon, the matron of St. Thomas's Hospital. The report says : "The committee cannot too strongly express their sense of the loss to the Nightingale Fund Training School which her resignation entails. She has uniformly fulfilled her duties so as to maintain the school in a high state of efficiency, and has earned the respect and esteem of all connected with it, and also, as the committee believe, with the hospital. The good wishes and gratitude of a long list of nurses will accompany her in her retirement." There is also an allusion to the death of Mr. William Rathbone, who is described as an ardent disciple of Miss Nightingale, "and one of the very first to give practical shape to the principles which she inculcated and the practice which she enforced." It is stated in the report that the annual gratuity of £2 allowed by the regulations for the term of three years to the nurses who have completed a year's service satisfactorily was awarded to 101 nurses. For candidates admitted after October 1st, 1900, these gratuities have ceased to be awarded, and it is intended that to those who have subsequently been admitted a certificate of training, upon conditions to be hereafter determined, will be granted. At the end of December, 1901, there were 50 probationers in the school at St. Thomas's Hospital, and 21 in St. Marylebone Infirmary. The balance of income over expenditure for the 12 months was £257 15s. 4d. We note that the latter includes a grant of £100 to the Metropolitan Nursing Association for District Training.

### THE NURSES OF BRISTOL ROYAL INFIRMARY.

A COMPLAINT which has been published in one of the local papers by a former nurse at Bristol Royal Infirmary, affirming that the work is excessive and the food insufficient, appears to have been answered by a present nurse in the infirmary, who gives a categorical contradiction of the statements. She declares that the number of nurses, in proportion to the number of patients, compares favourably with that found in any institution in England, and that the food is both good in quality and sufficient in quantity. Her reply is all the more impressive because she admits that when serious accidents come in there may "occasionally be a little extra work." To this no reasonable nurse would take exception. We learn from other sources that the Nursing Committee of the infirmary meet monthly for the sole object of overlooking the food and general comfort of the nursing staff; that the matron and assistant-matron always endeavour to arrange the diet of the nurses in order that they may have as much variety as possible ; and that, in fact, everything is done to ensure the comfort of the staff, which has been nearly doubled during the past five years.

### A COLLECTION REFUSED.

A CHURCH parade of cyclists was held at Alnwick recently, and it was proposed to give the proceeds of the collection, amounting to £14 15s. 6d., to the Alnwick and District Nursing Association. The

Duchess of Northumberland has, however, written to the representatives of the Association who attended the meeting of the organisers of parade, stating that she cannot authorise them to accept the generous offer, " as the Duke and herself much dislike this form of Sunday entertainment." It seems to us that this is an extreme course to take. The Duke and Duchess of Northumberland are obviously entitled to their own opinion about the propriety of Church parades of any kind ; but we do not see why they should impose it upon the Alnwick Nursing Association, even if they have contributed to that organisation a sum equivalent to the collection offered by the cyclists.

### MEMORIAL TO A ST. THOMAS'S NURSE.

There has just been dedicated by the Bishop of Chester in Capenhurst parish church a three-light window, representing an allegorical subject, as a memorial of the late Miss Helen Richardson, who died a year ago in Bombay. Miss Richardson was trained at the Nightingale School, and after leaving St. Thomas's Hospital undertook the work of a district nurse in the village of Chatteris, Cambridgeshire. In 1885, on the death of her father, the Rev. R. Richardson, of Capenhurst Hall, near Chester, she proceeded to Bombay, where she established an industrial home for women, and a rescue home. In her self-denying labours as a missionary of the Church in India, she found her training and experience as a nurse of great value.

### A HARD CASE.

When a nursing institution is managed by a committee there is generally a fair prospect of the nurses attached to it being treated satisfactorily. We cannot publish in detail the long letter which a nurse who has lately returned from Egypt sends us respecting her experiences this year in connection with an institution in Alexandria, but apart from the personal questions to which she alludes, a point of general importance is raised. She states that her agreement with the committee recited that there must be three months' notice on either side, except in the case of her misconduct, and also that a free passage to England would be given to her. Yet, when as the result of a controversy into which it is not necessary to enter, the committee wished her to leave, they requested her to go at once, though not alleging any kind of misconduct. Thereupon, she wisely consulted a solicitor, who communicated with the committee and required three months' notice on her behalf. This they declined to comply with, and offered, " without prejudice," a ticket to England if his client would leave forthwith on the French boat. As the nurse had not the means to fight out the matter, she reluctantly, against the advice of the solicitor, accepted the proposal, receiving only her salary to the date of leaving, instead of the three months' equivalent. We have repeatedly insisted upon the necessity of nurses taking the precaution to obtain written agreements both at home and abroad ; but if a committee decline to be bound by their own terms, the case is very hard indeed.

### A DEADLOCK AT AN IRISH INFIRMARY.

Some time ago the guardians of the Ballina Union decided to ask the Sisters of Mercy in the town to undertake the nursing in the workhouse infirmary. The Sisters consented on the understanding that they were invested with supreme control. But the guardians, instead of consulting the Local Government Board at the outset, postponed that step until they had arranged to grant the present matron a retiring allowance of £40 a year. It has since transpired that the Local Government Board are not prepared to sanction the pension, because they have no legal authority to do so, and as the matron will not resign until she is assured of it, there is a deadlock.

### A GIFT FROM MISS NIGHTINGALE.

Miss Florence Nightingale has shown her interest in the work of the new infirmary, Farnham, by presenting to it through the chaplain, the Rector of Farnham, silver plate for the Holy Communion for use in services held in the infirmary.

### INFIRMARY NURSES AND THE L.O.S. CERTIFICATE.

The importance of obtaining the certificate of the London Obstetrical Society is increasingly recognised by nurses. Four of the nurses of the Edmonton Union Infirmary have just passed the written and obstetrical examination and received the congratulations of the Guardians. Miss Franklin, the superintendent nurse, has also been congratulated upon the successful result of her painstaking instruction.

### AN OUTING FOR DISTRICT NURSES.

The annual outing of the nurses attached to the Suffolk Nursing Association has just taken place under very pleasant conditions. They were invited by Colonel and Lady Florence Barnardiston to visit Ryes, and 18 were able to accept the invitation, arriving between 11 and 12. The following districts were represented :—Ampton, Belton, Chelmondiston, Felixstowe, Ickworth, Icklingham, Kelsale, Lavenham, Livermere, Long Melford, Needham Market, Pakenham, Rickinghall, Stowlangtoft, Thurlow, Thurston, Westleton, and Woolverstone. The nurses of the other districts were prevented from attending. The Association medals were presented to five new nurses, and, favoured by fine weather, the company greatly enjoyed themselves in the beautiful grounds. A brief but inspiring address was delivered by the Rev. H. Barry, rector of Great and Little Henny, and the guests left after tea for their respective spheres of duty.

### WHY THE NURSES LEAVE CHAPEL-EN-LE-FRITH.

At nearly every meeting of the Chapel-en-le-Frith Board of Guardians the resignation or the appointment of a nurse is a matter of animated discussion. An inquiry by a member, who asked whether the head nurse could throw any light on the frequent resignations and changes, elicited the information that the chief complaint of the nurses was that " Chapel-en-le-Frith was so quiet, and there was nothing going on." This is a condition of things which we are afraid cannot be easily remedied. Nor is it by any means confined to Chapel-en-le-Frith. The "quietness" of many of the workhouse infirmaries is undoubtedly a reason why it is difficult for the guardians to retain nurses. We can only suggest that the more consideration there is on the part of the former, and the more sweet reasonableness on that of the latter, the less frequent will be the vacancies.

### Lectures to Nurses on Anatomy.

By W. Johnson Smith, F.R.C.S., Principal Medical Officer, Seamen's Hospital, Greenwich.

### LECTURE XXIV.—GLANDS AND THE FUNCTION OF SECRETION.

Living organisms of every grade exercise amongst their other vital functions that of forming from the constituents of the blood certain combinations, almost invariably fluid, which serve varied purposes such as (1) assisting some other physiological function of the same body; (2) relieving the blood of refuse and poisonous material; (3) affording nourishment to other bodies, especially the young of the same species, or (4) giving protection and help in the struggle for existence. As respective examples of these different purposes I may mention (1) the discharge of saliva which, as we have already learnt, is so beneficial in the function of digestion; (2) the steady flow of urine containing refuse material which if allowed to accumulate in the blood would give rise to the very grave symptoms of what is known as uræmic poisoning; (3) lactation, or the supply by the mammalian female of milk; and (4) the ejection of offensive and, in venomous forms of reptiles and other lower animals, of poisonous and, it may be, deadly fluids.

This function of separating material from the blood is termed *secretion* and the fluids thus separated are called *secretions.* A distinction is often made between those fluids which, like saliva and milk, serve a useful purpose, and those which, like urine, simply carry off useless and injurious material. The former are called *secretions*, the latter *excretions.*

The function of secretion, though quite beyond the direct control of the will, may be much influenced through the nervous system by mental conditions. Thus the flow of saliva may be accelerated by the sight and even the thought of savoury food, and be arrested during terror or anxiety; and lachrymation or the shedding of tears is often the result of deep emotion. Secretion is also influenced by changes in the general condition of the organism. The importance of inspecting the tongue in illness depends very much on this relation, the clean and moist surface of this organ in good health being due to free and healthy action of the salivary glands, and the dry and furred or dirty surface of the feverish patient to reduced or arrested flow of the salivary secretion. The hot dry skin of fever, and the dry rough and wasted skin of chronic and wasting disease are the results of impaired and disordered functions of the widely-distributed system of minute glands by which sweat and a fatty and lubricating discharge called *sebum* are secreted on the surface of the body.

The organs specially concerned in the function of secretion, and which separate from the blood, elaborate, and discharge at different parts of the organism the fluids known as secretions or excretions, are termed *glands.* These organs vary very much in size, in form, and in complexity. Some, like the sweat glands of the skin and the follicles of the intestinal canal, are of simple form and very minute; others, the liver and kidneys for instance, are large and very complex in structure. The common elements of all are a layer of minute cells which take the sole part in the office of secretion, and one or more *ducts* through which the secreted fluid is set free. The simplest form of gland is a simple tube, or blind pouch, lined by cells (Lieberkuhn's follicle, figs. 53 and 55 *a*). If we imagine a tube of this kind to be elongated and coiled we have the usual form of *sudoriferous or sweat gland* (fig. 55 *c*). If a number of tubes be collected together, each tube dividing and subdividing like an artery or vein and terminating in a coil we are presented with what is called a *compound tubular gland*, of which the kidney, in its minute structure, affords a good example (fig. 55 *d*).

Fig. 55.—Diagram showing structure of chief forms of glands—*a*, simple tubular gland; *b*, simple saccular gland; *c*, a simple tubular gland; *d*, a compound tubular gland; *e*, a racemose gland.

The most advanced form of gland structure is that presented in varying degree of complexity by those known as *racemose glands*, of which the pancreas, the mammary or breast gland, and the salivary glands are good examples. In this type of gland the blind ends of the ultimate subdivisions of each branched tube are dilated into rounded sacs or vesicles which are collected in distinct, though closely-united, groups called lobules, these lobules being clustered in the ramifications of the duct or tube-like grapes in a bunch (fig. 55 *e*).

Let us now briefly review some of the most useful and familiar varieties of secreting gland.

The discharge of sweat from the surface of the human body, which though only sensible or apparent in exceptional circumstances, is constantly being carried on during life in the form of what is called by physiologists insensible perspiration, is effected by a vast number of minute glands formed by simple coiled tubules, which are scattered over the whole surface of the body.

The clear and thin fluid which constantly moistens the front of the eyeball, and which in consequence of irritation or injury of the eye, or of painful emotion, is discharged in excess in the form of tears, is secreted by a lobulated or racemose gland—the lachrymal gland—of about the size and shape of an almond, situated under the roof of the orbit or eye-socket over the outer part of the eyeball.

Fig. 56.—Liver. Upper surface. A, right lobe; B, left lobe; C, gall bladder.

The liver (fig. 56), which is not only the largest gland but the largest and heaviest organ of the body, is placed, as we noticed in a previous lecture, in the upper part of the abdomen on the right side, where it is almost wholly covered in front by the lower ribs and corresponding rib cartilages. We should recognise this organ by its large and thick lobe, A, on the right side, and by the small and thinner

### LECTURES TO NURSES ON ANATOMY.—*Continued.*

left lobe, B, marked off from the former by a deep notch in front and a deep depression (longitudinal fissure) on its under surface. It is a lobulated gland, the lobules or secreting elements of which are closely packed together. These lobules secrete bile from the ultimate ramifications of the portal vein, which, as we have already learnt, is formed by converging veins derived from the intestinal canal, and discharge this fluid secretion into the smallest subdivisions of the special duct of the liver known as the hepatic duct.

The pancreas, known to most of us as "the sweetbread," is a racemose gland situated in the abdomen and extending from side to side over the front of the lumbar part of the spine and behind the stomach. Its secretion, which is discharged by a special duct into the duodenum, takes a very important action in the digestion of food, especially in promoting, with the aid of bile, the ready mixture or emulsifying of fatty matter with other constituents.

From the large liver and much smaller pancreas, both of which are single organs, we pass to the two kidneys placed almost symmetrically at the back of the abdomen on each side of the spine. Like other solid organs contained in the abdominal cavity they are well protected, being covered for most of their extent behind by the floating ribs. Each kidney is about 4½ inches in length and 2½ inches in breadth, and weighs, when healthy, about 4 ozs. In some very exceptional instances the two kidneys are forced into a single curved organ with the concavity downwards—the so-called horse-shoe kidney—a condition quite compatible with perfect health. The normal form of the human kidney is well known. It is curved in front, slightly flattened behind, has a convex external border, and a concave internal border corresponding to the spine, which border is marked by a deep notch called the *hilus*, to which are inserted the blood vessels (renal artery and renal vein), and below and behind these, the proper excretory duct of the kidney known as the *ureter*. A longitudinal section of the organ shows an external, well marked, and broad zone of secretory tissue called the cortical portion, and an internal layer arranged in the form of pyramids which project into branching divisions of a cavity (the pelvis) forming the dilated origin of the ureter or duct.

As its secreting tissue is made up of an elaborate system of coiled tubules, the kidney is to be regarded as a compound tubular, and not, like most of the other large secreting organs, as a racemose or compound saccular gland. In the performance of its special function the kidney acts as a filter, separating from the stream of blood constantly supplied by the renal artery and drawn off by the renal vein, a large quantity of clear fluid holding in solution a great variety of salts, of which common salt (sodium chloride) forms a large proportion, and, as its most important constituent, the poisonous waste product known as urea.

Whilst in most glands the function of secretion is performed intermittently, the elaboration of bile in the liver and the filtration of urine through the kidneys is carried on continuously, though at times these processes may be accelerated or retarded. As the continuous discharge of these fluids from the body would be either unnecessary or inconvenient, there is provided in each instance a special reservoir or bladder in which the fluid is stored up for a time until required for its special purpose, or until it has accumulated in excess. The *gall bladder* is attached to the under surface of the liver and projects forwards and downwards into the abdominal cavity at the middle of the thin lower margin of the right lobe (fig. 56 C). This reservoir communicates with a short duct—the cystic duct—about one inch in length, which joins a duct through which the bile is discharged directly from the liver. These two ducts, *the cystic duct* from the gall bladder, and the *hepatic duct* from the liver, join together to form a single duct—the *common bile duct*, which, after it has joined near its termination the duct from the pancreatic gland, opens into the duodenum.

The urinary reservoir or bladder is detached from the kidneys and forms a single bag or sac placed in the pelvis behind the *symphysis pubis*. This bladder, which is very distensible, and capable of containing, even under healthy conditions, from two to three pints of fluid, is connected with each kidney by the long and tortuous duct called the ureter, which opens above into the pelvis of the kidney as we have already seen, and below on both sides into the posterior and lower part of the reservoir.

### Three Months' Midwifery Training.

BY AN OCCASIONAL CORRESPONDENT.

I was anxious to get an appointment in the Colonies, and to that end put my name down on the Colonial Nursing Association list, but was told by the secretary that my chances of getting a post abroad were remote, as preference would be given to nurses holding the certificate of the London Obstetrical Society. The maternity hospital training schools were all full up, so I had to get my tuition privately. I was recommended to go to a midwife who took in pupils, and who had a large district in the south-east of London in connection with the York Road Lying-in Hospital. For the modest sum of 12 guineas I was to be provided with board, lodging, and practical instruction for as many weeks; for another guinea theoretical instruction was to be obtained at a trained nurses' club, and a very good collection of medical books and magazines was available for reference at the same place to members.

### No Sister to Go To.

I had had a year of gynæcological training in one of the big London hospitals, so I flattered myself that I should be quite *au fait* with the work, but I had not reckoned on the difference between nursing in a big ward, at regular hours, with every convenience at hand, and the makeshifts in squalid little rooms in the slums, with a fearful amount of responsibility on your shoulders, and no sister to go to for help or advice in emergency. There were three pupils besides myself and I was glad to find that I had a room to myself. Two of the others shared a room, and where the third slept was always a mystery to me. I believe she had an occasional nap in our rooms when we were out. My room was very small, and when my boxes were brought in there was hardly space to swing the proverbial cat in, but as I had no cat, and soon found out that even if I had one I should not have the time to indulge in gymnastics, I was quite contented with it. We breakfasted at 9 A.M. on bread and butter, bacon, sausages, or something of that sort, and tea. The food throughout my stay was excellent, well cooked, and plenty of it. After breakfast we were each handed a slip of paper on which was written our round for the day. Mrs. K. always accompanied the novices, so I was told to join her that day. I quite enjoyed the brisk walk in the fresh morning air, and felt equal to

### THREE MONTHS' MIDWIFERY TRAINING.—*Continued.*

any amount of work. I found afterwards that Mrs. K. very tactfully took new pupils to her best class of patients first, so we got accustomed by degrees to dirt and squalor.

### The First Case.

My first patient was a dear little woman, clean and tidy. It was her first baby, so with great pride she showed me her basket, well stocked with baby linen and all the etceteras for baby's toilet. Being a trained nurse I was able to get the antiseptics ready, and help with enemas, douches, etc., and I was also able to wash and dress the baby while Mrs. K. attended to the mother. Before leaving I was told to enter all particulars in my note book, which I had with me in my black bag. We had to enter name and age of mother, sex of child, which presentation (my first case was a breech), size of os when first examined, duration of labour, number of previous confinements, any abnormalities, temperature, pulse, and general condition of mother on leaving, etc. After the patient was made comfortable, we proceeded to several other houses lying mostly in the same direction, where there were babies to wash and dress, mothers to give douches to and tidy up generally, temperatures, etc., to be taken and duly entered in note-books. This was to be my "beat" for the next week or so, as every case was visited by us regularly for 10 days, and longer if necessary. As fresh cases took the old ones' places, new "beats" were formed, but Mrs. K. always endeavoured to let each nurse have all her cases in one direction, as far as could be managed, in order to save our legs.

### Shop Tabooed at Meals.

We were all mostly home by 2 P.M., and sat down to a substantial hot dinner. "Shop" was by common consent tabooed at meals, and very wisely too. There is nothing more exasperating and wearisome to my mind than the bad habit some nurses have of dragging in their work at all seasons. After dinner we had to get through the rest of our list. If we were lucky enough to get through in the morning we might reckon on an hour's rest, but not more, as Mrs. K.'s practice was very large, and sooner or later, generally sooner, the front door bell would tinkle, Barnes and Herman be reluctantly laid aside, and another round of afternoon calls started. Oh! that bell. How often had I just fallen asleep, to be roused by its unmerciful tinkle, followed promptly by Mrs. K.'s voice outside my door, "Wake up nurse, your turn for the next case," and then shivering with cold, and half asleep, plunge into my uniform and turn out into the raw damp November fog. Mrs. K.'s sister, who did the housekeeping, was very kind to the pupils. While we were scrambling into our clothes, and invoking blessings on babies and everything pertaining to them, she would light a spirit lamp and have a nice comforting cup of tea ready for us. Very often she would slip a packet of biscuits into my pocket, remarking, "Goodness knows when you will be back, better take something with you."

### The Slummier Part of the District.

Very soon I was taken to the slummier part of the district. It was my first introduction to actual want and misery, and the memory of it haunts me still. At first I used to go home and weep for hours over the wretchedness I was not able to alleviate. Anything I could do seemed such a tiny drop, quite lost in that terrible ocean of human suffering. The poor I had dealt with so far were in the colonies, and they were rich in comparison. They at least had God's blessed sunshine all the year round, the scantiest clothing sufficed them; and a few pence would provide a whole family with food for a week. Picture to yourself a tiny room, just big enough to hold a bed, there was nothing else, and scarcely room for one to turn. In the semi-obscurity lay the poor mother in the pangs of labour, with four small children in the same bed. Blanket or sheets there were none, just the bare mattress with a few dirty rags to clothe them. Although it was November there was no sign of fire in the grate, and not even a light. The gas was obtained by the penny-in-the-slot system, so I quickly got a light; then I took the children downstairs and asked a neighbour to look after them. After that I hastened to the nearest shop and bought a few penn'orths of coal and shavings to light a fire, and dispatched an urchin to get me a jug of milk. Soon I had the satisfaction of seeing a bright fire, the kettle of hot-water singing merrily, and some nice warm milk for the patient, who had not tasted food of any sort for over 24 hours. The confinement was a normal one, and the patient recovered in a wonderfully short time in spite of her surroundings. I had to have all my clothes baked when I got home, as the paper of the room hung in strips and, to my horror, I discovered swarms of insects in every hole. After that I generally left my bonnet and cloak in the cleanest room I could find in the tenement, or handed them to a child in the street, who took charge of them for a penny. The rest of my garments being of washing material did not matter so much, and, of course, my hair received the most scrupulous attention.

### The Value of Old Newspapers.

Mrs. K. advised me to take a roll of old newspapers when visiting the poorer patients, and I always found them most useful, even among the better class. Often where sheets and blankets were provided a mackintosh was never thought of, and my newspapers have saved many a bed from being stained. They have the advantage, too, of being easily disposed of when done with in the fire, and are certainly safer to use than a mackintosh. One poor mother had toiled very hard to have things nice and tidy, and was actually busy at the wash-tub when she was taken ill. Not only had she provided herself with clean linen, but the expected baby had a complete though modest outfit. Alas! poor mother, she had twins. She burst into tears when I gently broke it to her; it was very hard luck. They flourished amazingly, and were as sturdy a little pair as one could wish to see. I christened them Kitty and Jimmy, and provided Miss Kitty with a trousseau, including a christening robe. In order not to hurt her brother's feelings, I gave him a pair of blue sleeve-knots.

### Work in the Borough.

One night a messenger arrived hurriedly from the Borough. The case was urgent, so we had to start at once, at 2 A.M. The trams and 'buses had all ceased running for the night, so we had to tramp all the way, and climb about 15 flights of stairs at the end of our journey. I remember remarking to Mrs. K., after we had toiled up about ten flights, that if we went any higher we should probably be meeting the angels. On our return trip we had to fumble our way down all those stairs in Cimmerian darkness. At first these nocturnal visits rather alarmed me, but I soon got used to them, and found that my nurse's uniform was a greater safeguard than a police escort. I worked for three months in the worst parts of Walworth and the Borough, being out at all hours of the night, and never once had a rude word said to me. I must mention one little incident though. It was raining heavily so I donned my goloshes, and was trudging along with my cloak and skirts well tucked up, when a little boy suddenly stopped before me, stared hard at my feet, and said, "Lawks,

THREE MONTHS' MIDWIFERY TRAINING.—*Continued.*

sizes in shoes!" Now I am particularly vain of my feet, and I don't wear sixes, not even in goloshes, so I wanted to punch that boy's head, but he was too quick for me. That was quite the rudest thing I had said to me in that district.

Little Time for Theory.

We had excellent opportunities for acquiring the practical part of the work, but it left us very little time for theory. We attended lectures regularly twice a week in the afternoons, but had scarcely any time to write answers to the questions set us each week, so my Barnes and Herman were not worn to pieces. I have often wondered since, how untrained women manage to get through their midwifery training creditably. How do they find the time to acquire the hundred and one details of ordinary sick nursing which are essential to midwifery as well, such as temperature and pulse taking, the giving of enemas and douches, antiseptics, and even the making of a bed? I believe I am blessed with an average amount of intelligence, and yet I am certain that if I had not trained first as a sick nurse I should not have passed my midwifery examination, at any rate with satisfaction to myself.

The Examination.

I helped at 42 confinements during my three months' stay with Mrs. K., and as this was far beyond the number required by the Obstetrical Society, I was now allowed a little more time for reading up, as the exam. was approaching. The exam. was held at 9 P.M. We had a last coaching class early in the afternoon, and were told not to look at a book again, but take a stroll in the Park and then have a good dinner somewhere near Hanover Square. It was very good advice, as instead of worrying our brains over the problems to come we all turned up as fresh and bright as possible, and quite ready to face the whole board of examiners. The written exam. was held first, and I did not find any of the questions beyond me; in fact, when my paper was finished, I found I had three-quarters of an hour to spare. The *viva voce* was far more trying, but I was not nearly as nervous as I was at my preliminary nursing exam On that memorable occasion I was asked how many minims went to a drachm, and five precious minutes were wasted—we were only allowed ten minutes for the whole affair—before I could utter a word. This time I had fifteen minutes, and as I did not suffer from stage fright, we got through a good deal. At the conclusion I was handed a folded slip of paper "for the secretary." I was so anxious to learn my fate that I asked if I might look at it. The great man smiled assent, and as I saw the magic word "Passed" I felt rewarded for all the weary hours of toil I had gone through. A fortnight later I got my colonial appointment.

### Fever in the Milk-can.

A PERSONAL EXPERIENCE BY A LADY CORRESPONDENT.

I had gone to pay my milk bill, and as I took the receipt I noticed that my usually cheery dairy-woman was looking sad and worried, her cheeks pale, and her big brown eyes dim. "Are you well, Mrs. P.?" I asked.

Tears came with the answer: "I'm well enough; but my husband is to be taken off to the hospital to-day. He has enteric fever." I think she must have noticed the look on my face, for she added hastily, "You needn't be afraid, Mrs. S.; the milk doesn't come near the house." But I could not help recalling the fact that my cook had been out of sorts for some days, and had that morning gone home—getting the holiday I had promised her at Christmas a few days earlier—and that my little boy had been rather run down of late. Could it be that these two, who certainly were the members of the household who consumed most milk, were infected with the fever? I was sorry for the woman, but I was anxious on my own account, and I could not make up my mind whether or not I should go elsewhere for my milk. Next day the question was settled for me. The dairy was shut up by order of the sanitary authorities.

And next day my little son was laid down. The servant who had gone home hung about in a half-ailing condition for a few days, and then—on Christmas Day, to add to the irony of things—she too was pronounced to be suffering from enteric, and was taken to the hospital. Meanwhile, I heard of more cases everywhere. Within a few doors of my own home a mother and two daughters were laid up; next door to them the son and two daughters of the house were ill. All up and down our village there were cases, and in most houses where there was one case there were more—two, three, in one place four members of the same family were affected. The fever van from the hospital was seen every day, and the blue, brown, green, and grey cloaks of private nurses were more familiar objects than ever before. And in every case the patients were those who had got their milk from that one dairy. I heard many pitiful tales. In one case a child was taken to the hospital, not because there was lacking either means or accommodation to nurse her at home, but her mother was nearing her confinement, and the doctor thought the risk of contagion was too great. In another, a pregnant mother was taken away from her children, and neither she nor the baby ever came out of the hospital again. To add to the poignancy of this case, it happened that the woman was a second wife, and that her husband's first wife had died in exactly the same fashion. In a town one does not hear of other people, but in a village the affairs of one's neighbours, rich and poor, are inevitably brought to one's knowledge.

Meanwhile I was fighting for a life. How cruel it seemed, that sponging of the fevered little body with iced water! At first the child protested bitterly, but finally there was only a quick little moan and shiver at the first touch. And how keen was my disappointment when, within an hour, the thermometer again registered 104° or 105°. Then the difficulty of forcing milk and beef-tea down the throat of a patient who only asked to be allowed to drowse and drowse, and sleep his little life away. At the end of a week he became suddenly worse. The temperature ran down too fast; the heart was failing; the extremities getting cold; he was unconscious. "Give brandy," said the doctor. Yes; but how to administer it, when, though the mind was dormant the will was awake and that will was set against nourishment in any form? Even if you managed to force food or stimulant between those clenched teeth, it was spat out of th mouth indignantly.

The doctor resorted to nasal feeding. A second nurse came to assist the one who had borne the burden of the case so far, and every three hours it was my duty to hold the angry, resisting arms, while the soft rubber catheter was put up the nose and away down the throat too far to permit of the food being spat or coughed up again. As the catheter was inserted there was always an angry cry,

FEVER IN THE MILK-CAN.—*Continued.*

choking off into a discontented gurgle as the milk or beef-tea ran down the long tube into the throat, but after the administration was over there was only an irritable grunt or so before the coma became as heavy as before. And so the food fought the fever. At the end of a week the patient opened his eyes, though all the use he made of them was to note the preparations for each meal and utter a protesting whimper. He still refused to swallow. If we spoke to him he did not try to answer. Not the faintest smile rewarded one's attempts to talk to him; but the fever-bright eyes followed me as I moved about the room, and when the doctor bade him he would, unwillingly, but as if compelled by a superior will, put out his tongue for inspection. Presently that became his sole reply to any remark. Tell him that the day was cold, or wet, or fine, and the tongue appeared. Ask if he was well, or ill, or hungry—the eloquent though silent tongue made ever the same reply.

A word from the doctor, meant to be encouraging, a casual "perhaps" from one of the nurses, met an unacknowledged terror in my own mind. Would life be spared with reason taken away? My soul protested. I wanted to do something to save my child, yet what could I do? Just then came one of the nurses to ask me to help her in the nasal feeding. Perhaps she wondered why I said so sharply, "We must try to make him take his food by the mouth."

Well, it was a long and difficult business, but he did yield to open his mouth and let the milk go over the throat. And then I emphasised the point by saying, "That's better than having a nasty tube put up your nose." At the next meal there was some trouble, and the tube was resorted to, but this formed a sufficient basis of comparison. He found the natural mode of assimilation easier, and the tube was never required again.

But still he did not speak—did not try to speak. Was the power wanting, or the will? For a day or two every test failed to show which. But one day when I advised the day nurse to go for her walk before it grew too cold, she reminded me that I had not been out for several days.

"Never mind; I'm not going out to-day," I answered. I turned to our patient: "Willie, who is to stay with you—mother, or nurse?"

No word came in reply, but a white skeleton finger pointed to me.

"Nurse," I said, "the child wants to speak, and can't. I'm going to teach him the deaf and dumb alphabet." How eagerly he copied the signs as I made them, and how well he remembered them! No lack of intelligence there, thank heaven! Soon he could spell his own name—all our names—ask for anything he wanted. The thin eager fingers were never still. Some of his requests were funny enough. Asked if he wanted anything, he signed "Y-e-s" and quickly detailed his wishes: "Stamps, gramaphone, up." The last demand could not be complied with, but his stamp-album was soon brought, and the gramaphone set a-going. It was a Sunday afternoon, and our gramaphone records are mostly of a frivolous nature, but "my heart was inditing a goodly thing," and a psalm of praise was set to comic songs that day. He began to complain of the monotonous diet, and when asked what he wanted, worked out "minced beef, potatoes, jam-tart, dough-nuts;" an ideal meal for an enteric patient, certainly! But I told him an awful tale of a boy who had died through eating a currant cake too soon, and the doctor capped it with the tragic fate of another whose life had paid for a meal of sausages and potatoes. And after that the milk food went down without a murmur.

This dumb talk lasted for a day or two. Then one day, as he was fingering out words for his own amusement, I heard him make the sound of the letter as he formed it. It was only the faintest whisper but it was speech, and with a little trouble he was able to speak—only in husky, sibilant tones, it is true—but to speak. The next step came in laughter. A merry giggle came from the bed, and when I asked what was the cause of it, the reply came through the laugh full and clear, "Ian asked his father;" here the laugh was got under control, and in consequence the nature of Ian's question was told in an almost inaudible croak. But the trick of speech had been learned, and when I had made him practice saying "nurse" and "mother" while he laughed, he found again the proper position of tongue and vocal chords.

Then came the relapse—I had almost said the inevitable relapse—when the enfeebled frame had to fight a, happily, feeble attack of the fever. But of that I will not speak, since "all's well that ends well," and the day came when we lifted him from his bed, and clad him in garments of his elder brother's; for though his own were "a world too wide for the shrunk shanks," they were a world too short for them, so much had the child grown during his illness. And now the illness is a great and glorious recollection. He has told the harrowing tale of his sufferings to his special chum, who uses the information as a taunt to fling at his own younger brother. "You couldn't stand what Willie stood, Colin." And then follows the tale of the nasal feeding and the ice-cold sponge. "You couldn't bear all that."

"Yes, but I *beared* it," says Willie proudly.

*Magna est vanitas.* It is no insignificant weapon in the panoply of human courage.

### The End of the Coronation.

BY A NURSE.

It may interest your readers to hear something about the Special Service held in Westminster Abbey for the Colonial troops on Sunday last, 17th inst., from one of the privileged few who obtained admission to the Abbey on that occasion.

It was a scene never to be forgotten. I was on my holiday in North Wales on Coronation Day, so had no opportunity of seeing the procession; but I would rather have taken part in this service than merely have witnessed the procession from outside the Abbey.

My first impression was that the dear old Abbey had not been in the least spoiled by the transformation it had undergone. The seats are all so wonderfully arranged, one feels as if they had always been there. Nothing looks out of place, or destroys one's idea of the "fitness of things."

I was shown to a seat in what had been the Peers gallery, and thought the chairs extremely comfortable. They are in the shape of the old Chippendale chairs, and the seats are covered with white felt, tied on with white ribbon.

As I looked across at the opposite gallery, filled to overflowing with the friends and relations of the Colonials, I in some way realised the gorgeous scene it must have presented on Saturday, the 9th inst., when, at the moment that their Queen was crowned, the rank and beauty of England stood, and with one accord the peeresses placed their coronets upon their heads.

Below, and just in front of me, were the two thrones, on the costly carpet in the enclosed space reserved for royalty.

### THE END OF THE CORONATION.—*Continued.*

On all sides were curtains of embossed velvet, in rich hues of blue and old gold, the quiet colouring of which suited the ancient building better than any display of scarlet or purple. On my left hung a rare old piece of tapestry.

The choir were installed in the organ loft, and as the solemn tones of the organ pealed forth in the "Te Deum"—that grandest "Hymn of Praise"—one felt what an impressive scene it must be to those sons of the Empire for whose sake it was held, as a "God-speed" to them before leaving the shores of their mother-country—a fitting farewell from the mother-church.

The surroundings and trappings of royalty were all there, only royalty itself was absent; but the spirit of the great religious ceremony so lately enacted there was, I think, present to each one of us. Bishop Weldon gave a stirring and striking sermon, addressed entirely to those for whom the service was taking place, from the words, "Go home to thy friends, and tell them what great things the Lord hath done for thee." The soldiers were in the seats occupied on Saturday by the members of Parliament, and joined with hearty fervour in the hymns, "Soldiers of Christ, Arise," "Onward, Christian Soldiers," and the last and most beautiful, "O God, our Help in ages past." Then when the blessing had been pronounced, "God Save the King" was sung by the entire congregation, all standing, for the last time amid the Coronation surroundings of Edward VII.

The vast assemblage were in no haste to leave, and the officials in no wise hurried our departure. Many had friends and relations among the khaki-coloured uniforms, and the soldiers with their friends were all allowed inside the space sacred to Royalty. Great was the interest displayed as they stood gazing their fill at the old coronation chair and the golden canopy above it. I saw one man pass his fingers reverently over the woodwork, as if he felt impelled to touch the inanimate thing which was the symbol of so much power and might. I took one last look back, as I reached the other side of the organ screen and was walking down the nave. The vista through the arch was all of gold, for the lights above the altar illuminated all that part around the golden canopy and the thrones of crimson and gold. All else faded into misty vagueness in the shadowy distance of the venerable arches, and the dream was over—but it had been a golden one.

### The Naval Review.

### A NURSE'S IMPRESSION.

I reckoned myself a fortunate being when an invitation came for me to witness the Naval Review and I found myself at liberty to accept it. A sea trip in fine weather is always delightfully refreshing, and when to that trip is added a beautiful spectacle, no more charming pleasure expedition can be imagined. My only anxiety was the weather. The forecasts were distressingly pessimistic, yet I hardly dared think of the disappointment attending a stormy or rainy day. To my delight the day awoke to brilliant sunshine, with a warm and gentle breeze. The scene was gay on the Royal Pier at Southampton, where not only those joining boats for the Review but many spectators had collected. There would have been more present, but the docks afforded greater attraction; the ship carrying the Boer Generals had arrived there the same morning, and no one quite knew where they were, or if they might or might not be seen at any moment.

The steamer chartered by my friends lying alongside the pier was a spacious and rapid boat, and we were soon threading our way amongst the numerous craft around the pier and steaming ahead down Southampton Water. The low flat shores are not very beautiful, but there were many objects of interest afloat, and soon we were passing Netley Hospital, whose long line of red buildings looked imposing and stately. I was told by an army sister that the Queen's recent visit took them all by surprise, and, in consequence, she saw things just as they were without any preparation being possible. When the Queen went to Netley some time ago she was disturbed that her soldiers had not brighter quarters. Matters have been much improved since then. The War Office has done a great deal of redecoration, and a generous gentleman, Mr. Bischoffsheim, has sent new and comfortable beds, hung the corridors with pictures, and added to the number of easy chairs. Mr. Bischoffsheim did all this without any ostentation just as he provided an ambulance service for London. By the time our talk about Netley was ended the hospital was left far behind, and our attention was drawn to the rather deep bay at Cowes, where we knew the King's yacht was lying. We managed to identify it to our satisfaction, and then our eyes were turned to the stately avenues of ships we were approaching. We eagerly scanned our charts to learn the general arrangement of the Fleet, showing the torpedo destroyers in a long line lying just like a weird black dragon off the Portsmouth shore. Then we noted the two lines of battleships and cruisers; on the left hand those of the Home Squadron, on the right those of the Channel Squadron. The four foreign ships of course interested us especially. The Japanese with their strange black funnels striped with white looked very formidable. They are both of English build, but quite unlike any other vessel present amongst the English Fleet. The Italian and Portuguese vessels completed the foreign visitors. There was so much to see as we steamed down the lines that between admiration and an endeavour to learn all I could I became quite bewildered, and felt I had not used my eyes half enough, when the signal to clear the lines was made and we had to proceed to our moorings. Then the scene became panoramic and beautiful in the extreme. It was no longer possible to distinguish the lines; the vessels seemed round and about us without any fixed positions, caused by the swaying of the tide, which kindly varied the scene for us considerably. The sea was a beautiful dark blue; the turreted vessels seemed almost painted on its surface, and the sun shone on a bewildering variety of craft of all kind, some of which appeared very restive and fussy beside the stately ships which seemed to stand immovable. Watching the scene and discussing an excellent lunch, the time of waiting which we anticipated would be long, passed all too fast. Now our eyes were constantly turning towards Cowes, and yet the *Victoria and Albert* was well on her way before we definitely and at last identified her. She was standing out well from Cowes, preceded by two pioneer yachts the *Irene* and *Alberta*, when the guns commenced the Royal salute, which only ended just before the King's yacht entered the lines followed by the yachts *Osborne* and *Enchantress*. Then commenced the exhilarating sound of cheers from the manned warships, which was taken up almost without interruption from ship to ship throughout the Royal progress. Every now and then the strains of "God Save the King" made music across the water from the ships which carried bands. The scene was entrancing, and the air full of enthusiasm. The *Victoria and Albert* is so imposing that she was not dwarfed by the huge ships amongst which she passed. Some who knew more about correct ship lines than I did said her form was all wrong, but the uninitiated were content to be impressed by her fine proportions. The little *Alberta's* lithe form impressed one, as it always does, by its modesty. I could not help contrasting the occasion when I saw her last, following the sombre torpedo destroyers, through just such a line of mighty ships, bearing the remains of the great Queen to her last resting place, as the glorious winter sun went down. What a contrast! and yet, gay as the present scene undoubtedly was, the recent danger through which our Sovereign had passed made the welcome accorded him more than a merely light-hearted greeting. The spirit of thankfulness was in the air, and as just before we finally turned our back on the mighty avenues which represented England's greatest pride, the one final and united cheer rose in the air as the Royal Yacht reached her moorings, we all felt glad and thankful that all was well, and that the cloud of a nation's anxiety had been dispelled. Thus ended a beautiful day, a glorious scene, and a never-to-be-forgotten occasion.

### The Nurses' Bookshelf.

Nursing, General, Medical and Surgical. By Wilfrid J. Hadley, M.D., F.R.C.P., F.R.C.S. (London: J. and A. Churchill. 1902. 326 pp. 3s. 6d.)

The earlier part of this book deals with nursing pure and simple, and it can be cordially praised for its fulness of description and attention to detail. In this respect it compares favourably with many of the books which are now written for medical students, in which methods of treatment are frequently mentioned without any instructions as to the manner of carrying them out. In addition to the manipulations and services which strictly belong to the nurse, the minor operations of leeching, cupping, blistering, etc., are most fully described. The list of the symptoms of poisoning produced by an overdose of drugs which are in common use will be found very useful to the nurse. In the rest of the book an attempt is made to give nurses a general idea of the leading characters of the various diseases. This is always a difficult matter, as the selection of what to say and what to leave out is not an easy one, but Dr. Hadley has succeeded very well, particularly with the fevers, pneumonia and phthisis. In the matter of "treatment" he has not been quite so successful, for much that he writes would have been more appropriate if addressed to a medical student than to a nurse. There are, also, some important omissions; for instance, in the treatment of failing heart in mitral disease neither diuretics nor aperients are mentioned, and it is especially important for a nurse to observe whether the former really increase the quantity of urine, and whether the latter produce large watery motions, as they are intended to do. In speaking of rectal injections, the glycerine enema is omitted; and nurses are not told of the necessity for using the shortest possible nozzle (one which will not do more than pass through the sphincter) when they are employing enemata for the treatment of diseases of the rectum. Nevertheless, whenever special instructions are given to nurses, they are, for the most part, admirable. We would mention, in particular, the description of the methods of applying local treatment in skin diseases, the means to be adopted to prevent the dissemination of the disease in infectious cases, and the preparations to be made for a surgical operation. The short account of massage is very good, and it is a pity that the subject of electricity was not treated in the same way. On the whole the book is a most useful one.

### Appointments.

[No charge is made for announcements under this head, and we are always glad to receive, and publish, appointments. But it is essential that in all cases the school of training should be given.]

Alton Workhouse Infirmary.—Miss Beatrice White has been appointed staff nurse. She received her training and certificate at the Farnham Infirmary, where she also held the post of charge nurse. Miss Beatrice White holds the L.O.S. certificate, and also a certificate for massage.

Baguley Sanatorium.—Miss Emily Lowe has been appointed matron. She was trained at the London Hospital, Whitechapel, where she was subsequently staff nurse. She was also for a short time in charge of the infectious block, and has since been night superintendent of the Eastern Hospital, Homerton, where she took holiday duty for the assistant matron and housekeeper; and assistant lady superintendent at Monsall Hospital, Manchester.

Frimley Isolation Hospital.—Miss A. Alexander has been appointed matron. She was previously matron at the Newbury Infectious Diseases Hospital, the Luton Fever Hospital, and Colchester Fever Hospital. She was trained at the London Fever Hospital and the General Infirmary, Salisbury.

Royal Boscombe Hospital.—Miss S. E. Allsop, who has been appointed staff nurse, was trained at the Leeds General Infirmary.

St. Leonards Infirmary.—Miss Joan Inglis, previously matron of the Leeds Union Infirmary, has been appointed matron. She was trained at the London Hospital.

### Wants and Workers.

The matron of Woolwich and Plumstead Cottage Hospital, Shooters' Hill, S.E., will be glad if any kind friends will send old linen to her.

### TRAVEL NOTES AND QUERIES.

Switzerland from Aix-les-Bains (M. T.).—I do not think you could do better than somewhere round the Lake of Geneva. Send me a stamped and addressed envelope, and I will give you the name of a good and reasonable Pension in Lausanne, high above the lake. It is a private place, and so I must not name it in these columns. Lausanne is about 75 miles from Aix-les-bains. Then there is Chamonix to the east, about the same distance, but not quite so easy to reach, because the rail does not touch it. The position is very lovely, and you could live at the Hôtel de France et de l'Union for 6 francs per day, also about the same terms at the Hôtel Pension de la Terrasse.

Weather and Accommodation in the Channel Islands (A. R. C.).—I fear in such a year as this one cannot say anything very hopeful as to the first question. As a rule, weather is much the same as with us, only that it is considerably milder and there is more sun, but the same clothing is needed. Light woollen is the best and avoids the draggled look of cotton. Moreover by the end of October warm apparel will most certainly be needed. The hotels I quoted in Guernsey are not the first-class ones, as may be seen by their terms, but perfectly clean and nice. If you need really good rooms and the best of everything, I should recommend the "Old Government House," terms from 8s. 6d. per day, or "The Royal Hotel," same terms. Be sure to visit Sark if you do not stay there. I can warmly recommend the "Belair." If you stay in Jersey there is a choice of hotels. In St. Heliers "The Royal Yacht," 8s. 6d. per day, or "The Grand Hotel," 9s., "The Star" from 6s., and "The Weigh Bridge" from 5s. 6d. At St. Aubin "The Terminus Hotel," 6s., and "The Somerville" from 8s. 6d. St. Aubin is a very delightful spot in which to spend a week. Tell me if I can help you further.

### Everybody's Opinion.

**[Correspondence on all subjects is invited, but we cannot in any way be responsible for the opinions expressed by our correspondents. No communication can be entertained if the name and address of the correspondent are not given as a guarantee of good faith, but not necessarily for publication. All correspondents should write on one side of the paper only.]**

### NURSING AMONG THE POOR.

"L. O. S." writes: I was much interested in reading the experiences of a pupil midwife in your issue of the 9th inst., and was very forcibly reminded of the time when I was training. I should like to suggest to those intending to take up this work that they make a small collection amongst their friends before starting. If I had my time over again I should most certainly do so, as it is in such work that a nurse finds herself in a position to help where help is needed, and that without pauperising the poor. When I was training I remember how I used to envy a nurse with whom I was working. She had private means with which to help these poor suffering women, and often by spending quite a small sum was able to send for such things, which were perhaps not an absolute necessity, but at least added greatly to the immediate comfort of the patient. In all the work that I have done amongst the poor, which has been in the most crowded parts of London, two things have constantly been brought before me—firstly, the great pluck they have; and secondly, the large amount of gratitude they show. I am convinced that nursing amongst the poor is by far the most noble part of our profession, but it is not every nurse who can see her way clear to doing this work, neither would everyone be adapted for it. I, too, remember going out one very bitter morning about 4.30 A.M., but instead of 12 hours it was 24 hours that we sat with that poor creature in a very tiny, little, stuffy room, and, while looking to our patient, had to keep a sharp look-out on the mother who was quite tipsy and worse than useless, and who, at our request to leave her daughter, utterly refused to do so. I think it is the gratitude of these people that helps a nurse along. When she is feeling tired and dragged to bits with long hours and hard work, she feels that it is worth it all if by doing so she has helped one of the world's less fortunate ones over a stile. There is a great deal, too, which appeals to one's humour in this work—the quaint sayings and ways of the people, and there is also a never-ending scope for the power of adaptability to be brought into use. How well I remember on one occasion wanting a jug, of which the dresser looked full, but lo, to my astonishment, when I took them down one by one, none of them would hold water; but all the wounded sides had been discreetly turned from sight and gave the appearance of comparative wealth. No one should take up this work, nor indeed nursing at all, unless they have a certain sense of humour, for it helps more than anything else to brighten their own lives and consequently the lives of those around them; and when the nurse meets them every morning with a smile and something pleasant to say, they look forward to seeing her as the chief feature of the day, and even look back upon the time when baby came as one of comfort and pleasure. Personally, I look back upon my training as an enjoyable time, and one that opened my eyes considerably to the way in which some of the poor live and the difficulties they have to contend with. If some of the people who talk so much about the "thriftless poor" were but to peep into these homes, I think they would feel as I did, that were I put in their place I should not be able to manage half so well as they.

### MATRONS AND PROBATIONERS.

"Sister" writes: With regard to the many complaints we constantly hear of the private nurse, are the matrons and sisters of our hospitals quite blameless? I feel that with thirteen years' experience in hospital I have some knowledge as to the training of probationers. The majority of women who enter the nursing profession now are from the educated and refined classes who realise that hospitals are not luxurious institutes for their convenience, but that they offer in return for the probationer's services to train them in the details of sick nursing. Let me give an instance of very common occurrence in our hospital—I hear it is the same in some others. A probationer comes for a month's trial with the distinct understanding that at the end of that time, if suitable, she shall stay for her two or three years' training. The trial being satisfactory she is accepted, and works on for six or twelve months, when for some trifle or personal dislike of a matron or sister she is told that the matron considers her unsuitable for further training, and forthwith she is either dismissed at once or given a month's notice, though this is a distinct breach of the agreement under which the probationer enters. Moreover, is it just to the institute which has given board, lodging, uniform and training and the more conscientious officers who have given the training to have the probationer discharged at a time when she begins to repay them for their work? Is it policy that the public should know that certain hospitals only half train their nurses? The result in our hospital is that immediately a probationer is treated so she obtains a copy of The Hospital and applies for the post of assistant nurse in a nursing home where the salary offered is from £20 to £25. I know two large nursing homes where they advertise for fully-trained nurses, salary £35 to £40, who constantly take our probationers with under a year's training. A probationer who secures a post, in a few days announces to the matron her desire to leave the next day. Various objections as to the difficulty of obtaining work, threats of no reference, &c., are of no avail. The probationer, seeing the extent to which injustice will go, is not convinced to study hospital's convenience. Consequently the staff is short, the other pros. are overworked and often for a very considerable time, in which often one after another fall ill. In spite of many advertisements, "Probationer wanted immediately," they seem difficult to get. Would it not be more loyal if after taking a probationer matrons would fully train her to the very best of their ability instead of turning her half-trained to serve the public? If some matrons would consider the future, and the danger of the half-trained nurse, if only as far as it affects the profession, I think there would be fewer complaints of private nurses. Having been trained under a strict but very just matron, I stayed five years after my training, and I sympathise with the pros. of to-day. In no way was I a model pro., and in no way had I an easy time, but I had a very happy one. To lose a monthly day off duty, or to be suspended from duty for a day was sufficient to bring the most buoyant spirit into subjection, besides giving all sides time for consideration. A probationer is not always all to blame, and a matron in order to gain real respect must be just. The trial month should be the trial, and after that both sides should be just and make the best of the agreement.

### PRIVATE NURSING.

"Home Sister," writing in reply to "Inquirer," would be glad to know whether in the statement, "the nurse was sent away at a moment's notice because the case only lasted two weeks," we are to understand that the nurse was dismissed from a home she was attached to, for that reason only? And does the expression, "the matron got the quarantine fee" mean that a home which undertook to supply nurses for infectious work had no proper accommodation for their isolation afterwards, and that the nurse was hereby compelled to risk carrying infection over any unlimited area she might have to traverse before finding a suitable place? And was it the same matron who again employed her at the end of two weeks? Also is not £4 4s. a week rather a rare fee for a case out of the metropolis? To anyone with a knowledge of homes, such statements are not easy of acceptance.

### To Nurses.

We invite contributions from any of our readers, and shall be glad to pay for "Notes on News from the Nursing World," or for articles describing nursing experiences, or dealing with any nursing question from an original point of view. The minimum payment for contributions is 5s., but we welcome interesting contributions of a column, or a page, in length. It may be added that notices of appointments, entertainments, presentations, and deaths are not paid for, but that we are always glad to receive them. All rejected manuscripts are returned in due course, and all payments for manuscripts used are made as early as possible after the beginning of each quarter.

### Echoes from the Outside World.

### Movements of Royalty.

THE departure of the King from London last week was made the occasion of a public demonstration. It had been announced that the royal party would approach Victoria Station by what is now called the new processional route, Constitutional Hill and Grosvenor Gardens, and enormous crowds assembled everywhere. Great interest was, of course, taken in the arrival of the Prince of Wales' children, who came to bid good-bye to their grandparents, and afterwards assembled on the balcony to see the departure of the little procession. The King, who was accompanied by the Queen and Princess Victoria, drove in a carriage and four with postillions and outriders, and looked well, although the fatigue and excitement had evidently somewhat tired him. The journey to Portsmouth was accomplished without incident, and the royal yacht came to her moorings at Cowes at six o'clock on Thursday. In the evening His Majesty, accompanied by the Prince of Wales, paid a visit to the Empress Eugénie on board the *Thistle*, remaining nearly half an hour, and afterwards he received all the officers aboard the royal yachts and presented them with Coronation medals. The following day the King, on a steam barge, repaired to the *Alberta*, and laid on the deck, between the royal pavilion and the bridge, a brass cross bearing the following inscription:—"V.R.I. Here rested the beloved remains of Queen Victoria from February 1st to 2nd, 1901. Born May 24th, 1819. Died January 22nd, 1901." It will be remembered that the *Alberta* conveyed the body of Queen Victoria from Cowes to Portsmouth at the time of the funeral.

### The King and the Fleet.

THE review of the Fleet by the King at Spithead on Saturday closed the Coronation festivities in a brilliant and fitting manner. Fortunately, the weather was favourable, and many thousands witnessed the ceremony both on sea and shore. At eight o'clock the Fleet was dressed with flags; the sight was then exceedingly pretty, as the long lines of black war ships were suddenly made gay with bright bunting fluttering in the breeze. There was no smoke to mar the spectacle, an order having been given that only Welsh coal was to be burnt on the steamers. Huge liners containing members of both Houses of Parliament, the Colonial Premiers, and other personages of note, put in an early appearance. At half-past two the King left Cowes, his yacht steaming slowly through the Fleet. As she passed each ship the officers and crew cheered His Majesty, who was very punctilious to acknowledge the salutes accorded him. The Queen, the Prince of Wales, and the Princesses were all distinctly visible. At a signal from the *Royal Sovereign*, after the *Victoria and Albert* had dropped anchor in her allotted place, the whole fleet cheered together, the cheers being led off by the Commander-in-Chief. So deafening was the sound that it was heard on Southsea Beach and far down the Solent. The fleet was magnificently illuminated at night.

### The Aftermath of the Coronation.

WESTMINSTER ABBEY has been thronged with visitors ever since Wednesday of last week. On that day admission was 5s., but so numerous were the people who wished to inspect the historic scenes of the 9th, that £1,340 was received in admissions. A wait of two hours was necessary to gain admittance, and a large force of police was present to see that persons kept their proper places in the queue which extended as far as Palace Yard, Dean's Yard and other places in the vicinity being filled with the carriages of those who had taken up their position in the crowd. The famous blue carpet was laid along the choir on each side of the nave, so that although the visitors could not walk, they could gaze upon it. The sacrarium was lighted up all day, on what is termed the "Theatre" were placed the Thrones of the King and Queen, and behind stood the ancient Coronation Chair, with the Stone of Destiny showing plainly; the canopy of cloth of gold was also exhibited. On Thursday the price of admission was 2s. 6d. only. Friday and Saturday the fee was reduced to 6d. On these days the queue extended a mile in length, as far as Bessborough Gardens, Vauxhall, and the authorities decided that the Abbey should also be opened on Monday and Tuesday this week. On Sunday a special service for Colonial troops was held at eleven, and the general public were not admitted.

### Presentation to General Baden-Powell.

A SOMEWHAT unusual presentation by proxy was made last week by Mr. Seddon, the Premier of New Zealand. More than 6,000 Civil Servants of the colony had subscribed sufficient in shilling subscriptions to purchase a solid gold dish in recognition of General Baden-Powell's bravery in successfully defending Mafeking against the Boer invasion. This dish had been brought to England by Mr. Seddon and his wife, and they had hoped to have delivered the offering to the General in person, but unfortunately he is still absent in South Africa, where he is organising the South African Constabulary. But Mr. Seddon communicated with General Baden-Powell with respect to the tribute, and he received the following cablegram: "Hearty thanks, please hand gift to my mother." The dish bears emblems of New Zealand scenery and life, and has this inscription:—"Presented by the members of the public service of New Zealand to Major-General Baden-Powell, a recognition by them of his and the garrison's gallant and successful defence of Mafeking, May 17, 1900." Then in the Maori language follow the words, "Oh the brave! Your actions are heard of everywhere." Mrs. Baden-Powell, in replying to some appropriate words of Mr. Seddon's, stated that she accepted with great pleasure the care and charge of the magnificent gift, and she trusted that it would be handed down to future generations as a souvenir of the friendly and loving feeling between the Colonies and the Mother Country, and especially New Zealand's appreciation of honourable deeds.

### The Boer Generals in England.

ON Saturday Generals Botha, Delarey and De Wet arrived at Southampton in the *Saxon*, and were escorted to the *Nigeria*, where they were received by Mr. Chamberlain, Lord Roberts, and Lord Kitchener. General Botha was attired in a blue pilot suit and a soft grey felt hat; General De Wet in a grey tweed suit and a brown hat; and General Delarey in a dark serge suit and a hard round hat. The Colonial Secretary seemed particularly interested in General Botha's little son, a boy of ten, and chatted to him for several minutes. The whole party spent some time on the upper deck of the *Nigeria*. The Boer generals subsequently proceeded to London by the boat express. At Waterloo a large crowd had assembled, and the saloon carriage in which the Generals travelled was besieged by the spectators. They responded to the enthusiasm displayed by raising their hats, but declined to make any statement except that they were much gratified at their reception in England.

### The Deanery of Westminster.

THE King has accepted the resignation of the Dean of Westminster, which will take effect on September 29th. Dr. Bradley, who succeeded Dean Stanley, has held the position for upwards of twenty-one years, and he is now in his 81st year. His health has been failing for some time, and he decided some time ago to retire after the Coronation.

### For Reading to the Sick.

"SHOW ME THE WAY."

Show me the way that leadeth unto Thee:
Though it be difficult, Thou art all might,
Though low, Thou art of love a boundless sea
Though dark, Thou art Thyself the living Light,
Though toilsome, Thou art goodness infinite,
And wilt refresh the heavy-laden soul
That comes to Thee; guide me to Thee aright;
I cannot come unless Thou dost control;
Lead Thou, enlighten, draw, and fill my being whole.

. . . . . . . .

May I be lost in Thy great Majesty,
Myself no more, to have no cherish'd thing,
No chance, no hope, no sorrow but in Thee,
My Shepherd, and my Father and my King!

*Isaac Williams.*

---

O Lord Christ, who hast said, Take heed what ye hear, how ye hear; give us grace to cast all our care on Thee who carest for us.

Because Thou hast called us, give us grace to obey Thy call.

Because Thou showest us wonderful things in Thy righteousness, give us grace to worship Thee trembling.

Because they that fear Thee lack nothing, give us grace to fear Thee.

Because they who seek Thee shall want no manner of thing that is good, give us grace to seek Thee until we find Thee.

Because Thou hast loved them that loved Thee not, give us grace to love Thee. Amen.—*Christina Rossetti.*

---

We are in the hands of One who watches for our good, and who knows when to grant and when to refuse our wishes. When we understand this truth, life becomes easier. We know that all things work together for good. We know that when our prayers are offered we can rest. We know that it is "goodness still which grants it or denies." We know more: we know that the wisdom and love which order all can grant by seeming to deny, can grant in some nobler way or in some other world.

*Bishop Boyd Carpenter.*

---

The Lord knows how to make stepping-stones for us of our defects, even; it is what he lets them be for. He remembereth—He remembered in the making—that we are but dust; the dust of earth, that He *chose* to make something little lower than the angels out of.—*A. D. T. Whitney.*

---

O dearest Lord! to feel that Thou art near
Brings deepest peace and hushes every fear;
To see Thy smile, to hear Thy gracious Voice,
Makes soul and body inwardly rejoice
With praise and thanks.

Thou reachest down to us Thy wounded Hand,
And at Thy Cross, dear Lord, ashamed we stand,
Remembering all Thy truth through weal and woe
Until our eyes with tears must overflow
Of thanks and praise.

*From "Lyra Germanica."*

### Notes and Queries.

**The Editor is always willing to answer in this column, without any fee, all reasonable questions, as soon as possible.**

**But the following rules must be carefully observed:—**

**Every communication must be accompanied by the name and address of the writer.**

**2. The question must always bear upon nursing, directly or indirectly.**

**If an answer is required by letter a fee of half-a-crown must be enclosed with the note containing the inquiry, and we cannot undertake to forward letters addressed to correspondents making inquiries. It is therefore requested that our readers will not enclose either a stamp or a stamped envelope.**

*Advice.*

(126) I am a trained nurse, and think of taking up work privately in the country. Will you kindly tell me if you think it would be etiquette to write and ask the various medical men in the district if they would grant me an interview, at the same time enclosing stamps for a reply, or would it be better to call and take my chance of seeing them without writing?—*Nurse.*

It would be quite right to ask for an appointment beforehand, but if you could find out the hours when the doctors are at home and then call, it would save both parties considerable trouble, and perhaps have better results.

I am a qualified dispenser, and I should like to go abroad to India to a public or a private appointment. I fear that I ought to have a nurse's certificate in order to succeed. Would you advise me to give up my present appointment worth £78 per annum and go in for a three years' certificate? I love nursing for its own sake, but I found the work at a large hospital rather trying.—*An Inquirer.*

You would certainly make a mistake to throw up a good post for an uncertainty.

I am in charge of a mental case (a ward in Chancery). She has rooms at the top of the house, and the food supplied her is very poor and so insufficient that I am obliged to supplement it or we should both starve. The sum of £200 a year is paid for us. Will you kindly tell me to whom I ought to complain?—*Justice.*

Write to the Secretary, the Lunacy Board for England and Wales, 66 Victoria Street, London, S.W.

Can you tell me of any special place in England, seaside preferably, where there would be a good opening for a maternity and surgical nursing home? Is such an institution needed in Eastbourne or Ilfracombe? What would be the best way of going about to establish it?—*F. S. W.*

This is a matter on which we cannot give advice. So much depends upon circumstances.

I have completed my training, and am thinking of starting private nursing on my own account in my own town, as there is a good opening. Will you kindly advise me how to word my card? I am preparing to take all kinds of cases, medical, surgical, and fever.—*Nurse.*

Have an ordinary lady's visiting card with your qualifications underneath the name; then have a second card with your terms for the various classes of cases, and the names of the medical men who will recommend you.

I am about to start a midwifery district practice where the doctors are unknown to me. Would it be correct for me to call upon them and say what I am going to do?—*L. O. S.*

Yes, it would be quite correct; but is it necessary?

*Maternity Case.*

(127) Will you kindly tell me of a maternity hospital in London where a patient can be received by paying a small sum?—*Nurse Hall.*

The British Lying-in Hospital, Endell Street, St. Giles, W.C., and the Clapham Maternity Hospital, 41 Jeffrey's Road, S.W., both make arrangements for paying patients.

**Standard Nursing Manuals.**

"Nurses' Dictionary of Medical Terms." Cloth, 2s.; leather, 2s. 6d.

"On Preparation for Operation in Private Houses." 6d.

"Hospital Sisters and their Duties." 2s. 6d.

"Medical Gymnastics, including the Schott (Nauheim) Movements." 2s. 6d.

"The Human Body." 5s.

"Practical Handbook of Midwifery." 6s.

"A Handbook for Nurses." (Illustrated.) 5s.

"Tendencies to Consumption: How to Counteract Them." 2s. 6d.

"Syllabus of Lectures to Nurses." 1s.

## Figures and Tables

**Fig. 55. f1:**
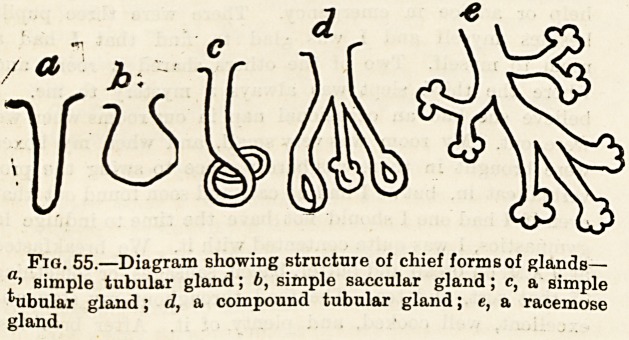


**Fig. 56. f2:**